# Longevity Studies in the New Normal: The Move to Virtual Assessment

**DOI:** 10.1093/geroni/igab046.526

**Published:** 2021-12-17

**Authors:** Stacy Andersen, Sandra Rizer, Lance San Souci, Melissa Berlin, Emily Harris, Stephanie Cosentino, Paola Sebastiani, Thomas Perls

**Affiliations:** 1 Boston University School of Medicine, Boston, Massachusetts, United States; 2 Columbia University Irving Medical Center, New York, New York, United States; 3 Columbia University Medical Center, New York, New York, United States; 4 Tufts Medical Center, Physician Organization, BOSTON, Massachusetts, United States; 5 Boston University, Boston, Massachusetts, United States

## Abstract

Extreme longevity is associated with resilience to Alzheimer’s disease. A major goal of centenarian studies is therefore to identify factors associated with maintaining cognitive function throughout life. Over the past year, two studies of centenarians and their offspring (age 60-110+ years) have pivoted from in-home assessments of cognitive and physical function to hybridized, Zoom-based assessments including comprehensive cognitive testing, blood pressure, grip strength, and accelerometry and biological sample collections. Protocols were optimized for accessibility for individuals with limited technology experience (e.g., investigator remotely controls all functions of the participant’s tablet) and sensory impairments (e.g., integration of wireless headphones) and include high-sensitivity data collection (e.g., sensor-based wearables and digital recording of cognitive test responses). Advantages of virtual administration included the ability to accommodate fatigue through multi-day assessment and to include geographically-isolated individuals. Disadvantages included participant burden due to equipment setup and inability to collect certain measures virtually (e.g., carotid ultrasounds).

